# Effect of a Real-Time Artificial Intelligence-Assisted Ultrasound System on BI-RADS C4 Breast Lesions Based on Breast Density

**DOI:** 10.3390/cancers18030536

**Published:** 2026-02-06

**Authors:** Jeeyeon Lee, Won Hwa Kim, Jaeil Kim, Byeongju Kang, Joon Suk Moon, Hye Jung Kim, Soo Jung Lee, In Hee Lee, Ho Yong Park

**Affiliations:** 1 Department of Surgery, School of Medicine, Kyungpook National University, Daegu 41566, Republic of Korea; j.lee@knu.ac.kr (J.L.); libertas033@knu.ac.kr (B.K.); joonsukm@knu.ac.kr (J.S.M.); 2 Kyungpook National University Chilgok Hospital, Daegu 41404, Republic of Korea; greenoaktree9@gmail.com (W.H.K.); mamrad@knu.ac.kr (H.J.K.); sj.lee@knu.ac.kr (S.J.L.); ihleeoncology@knu.ac.kr (I.H.L.); 3 Department of Radiology, School of Medicine, Kyungpook National University, Daegu 41566, Republic of Korea; 4 BeamWorks Inc., Daegu 41404, Republic of Korea; jaeilkim@knu.ac.kr; 5 School of Computer Science and Engineering, Kyungpook National University, Daegu 41566, Republic of Korea; 6 Department of Oncology, School of Medicine, Kyungpook National University, Daegu 41566, Republic of Korea

**Keywords:** breast, BI-RADS C4, artificial intelligence, ultrasound

## Abstract

Background: Artificial intelligence-based computer-aided diagnosis (AI-CAD) systems are increasingly used in breast ultrasonography; however, their diagnostic performance may vary with breast density. Given that dense breasts are highly prevalent among Asian women, understanding this relationship is essential for optimizing AI-assisted imaging strategies. Therefore, this study aims to evaluate the effect of breast density on the diagnostic accuracy of an AI-CAD ultrasound system in BI-RADS category 4 (C4) breast lesions. Methods: Overall, 110 consecutive BI-RADS C4 lesions were reviewed between January and December 2023. An AI-CAD ultrasound system automatically assigned BI-RADS categories and calculated the probability of malignancy (POM) using static ultrasound images. Histopathology served as the reference standard, with atypia and malignancy combined into a non-benign category. Diagnostic performance—including sensitivity, specificity, positive predictive value (PPV), negative predictive value (NPV), and overall accuracy—was analyzed based on breast density (BI-RADS B–D), determined using AI-assisted mammography. Results: Overall, the sensitivity and NPV were 81.3% and 87.5%, respectively, while the specificity and PPV were lower at 53.8% and 41.9%. All diagnostic performance metrics improved with increasing breast density. In the density D category, sensitivity (92.3%), specificity (61.5%), NPV (96.0%), and accuracy (69.2%) were highest. Additionally, concordance between AI-assigned BI-RADS categories and histopathologic diagnoses increased with density (B: 50.0%, C: 57.5%, D: 67.3%). Across all density groups, non-benign lesions consistently demonstrated higher POM values. Conclusions: Breast density significantly affects the diagnostic performance of AI-CAD ultrasound in BI-RADS C4 lesions. The AI system demonstrates higher accuracy and concordance in dense breasts, suggesting more consistent lesion interpretation in high-density environments. These findings highlight the potential utility of AI-assisted ultrasound as a diagnostic adjunct, particularly for Asian women, who commonly have dense breast composition. Further multicenter, real-time validation studies are warranted to validate these findings.

## Simple Summary

Breast ultrasound is widely used to evaluate suspicious breast lesions, particularly in women with dense breasts, but many BI-RADS C4 lesions ultimately prove to be benign and still undergo biopsy. Artificial intelligence (AI)-assisted ultrasound systems have been developed to support diagnostic decision-making, yet their performance may vary according to breast density. In this study, we evaluated an AI-based ultrasound system in 110 BI-RADS C4 breast lesions and analyzed its diagnostic performance across different mammographic breast density categories. The AI system showed higher sensitivity, specificity, and overall accuracy as breast density increased, with the best performance observed in extremely dense breasts. Conversely, lower-density breasts showed more false-positive results, likely due to heterogeneous background tissue. These findings suggest that breast density is an important factor influencing AI performance in breast ultrasound. AI-assisted ultrasound is useful as a decision-support tool for reducing unnecessary biopsies in women with dense breasts.

## 1. Introduction

Artificial intelligence (AI) is rapidly expanding its applications in medicine [1,2,3,4]. In radiology, combining AI with computer-aided detection and diagnosis (CADe/x) technologies serves as a powerful diagnostic tool for enhancing diagnostic accuracy and efficiency [5,6,7,8]. In breast disease, AI-based diagnostic support systems are increasingly integrated into clinical practice alongside various imaging modalities, such as mammography, ultrasound, and magnetic resonance imaging (MRI) [9,10,11]. Representative examples include Lunit INSIGHT MMG^®^ (Lunit, Seoul, Republic of Korea) for mammography and CadAI-B^®^ (BeamWorks Inc., Daegu, Republic of Korea) for breast ultrasound, highlighting their clinical utility [12,13,14]. However, the extensive analytical output generated using AI introduces limitations because its performance can vary significantly depending on the interpretation of the clinician and the specific context of application.

Breast ultrasound is widely used for diagnosing breast diseases because it involves no radiation exposure, provides real-time imaging, and enables detailed assessment of lesion location, size, and morphology. However, mammography remains the standard modality for population-based screening, and ultrasound is typically used as a complementary tool because of its operator-dependent nature and variable diagnostic performance. In particular, BI-RADS category 4 lesions represent a major diagnostic challenge, as biopsy is routinely recommended despite a high proportion of benign outcomes.

Recent advances in artificial intelligence have led to the development of AI-based computer-aided diagnosis systems for breast ultrasound, primarily aimed at improving diagnostic consistency and supporting biopsy decision-making. However, most prior studies have evaluated AI performance across heterogeneous BI-RADS categories or through binary benign–malignant classification, providing limited insight into AI performance at the clinically critical BI-RADS 4 decision threshold. Moreover, breast density—an important factor influencing ultrasound image contrast, lesion conspicuity, interpretability, and image-acquisition parameters such as time gain compensation—has not been sufficiently considered in prior AI ultrasound studies. Additionally, it alters image-acquisition parameters, such as Time Gain Compensation adjustment [15,16]. Therefore, breast density should be considered a fundamental factor when evaluating the diagnostic accuracy of AI-CAD in breast ultrasound, as it can significantly influence system performance.

Given that 50–70% of Asian women have dense breasts [17,18,19] and breast cancer occurs approximately 10 years earlier in Asian populations than in Western women, AI-based breast ultrasonography may serve as a more widely applicable and effective supplemental screening and diagnostic tool in this population [20,21,22,23]. Although AI-based breast ultrasound systems have been increasingly evaluated for their overall clinical accuracy and utility, evidence regarding their diagnostic performance in relation to breast density remains limited. This study aims to specifically investigate the impact of breast density on the diagnostic performance of AI-CAD ultrasound examinations.

## 2. Methods

### 2.1. Study Population

**Figure 1:**
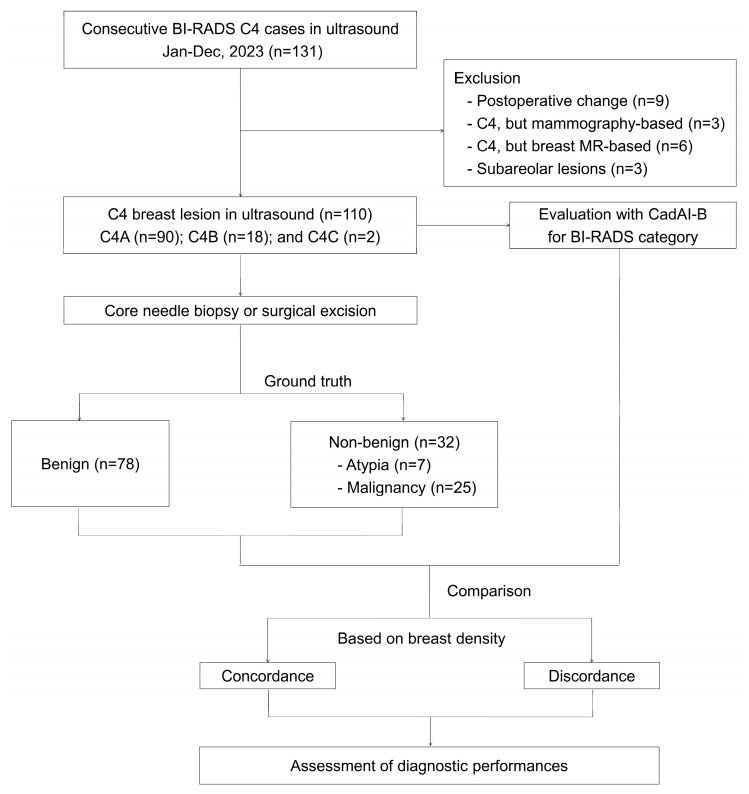
Study design of AI-CAD-assisted breast ultrasound analysis in BI-RADS 4 lesions stratified by breast density.

Between January and December 2023, 165 consecutive patients undergoing breast ultrasonography for suspected breast disease and subsequently classified as American College of Radiology Breast Imaging Reporting and Data System (ACR BI-RADS) category 4 (C4) were evaluated [24]. Only lesions classified as BI-RADS C4A–C by board-certified breast radiologists were included. During screening, several cases were excluded for the following reasons: the lesions were visible only on panoramic ultrasonographic images; the key representative image contained a caliper that interfered with AI processing; no static images were captured or saved for analysis; or the patient declined needle biopsy despite clinical recommendation. Additionally, postoperative cases and lesions with mammography or breast MRI performed before ultrasound—potentially influencing interpretation—were excluded to ensure consistency in imaging-based classification. Therefore, the final study population comprised exclusively ultrasound-based C4 lesions with valid static images and confirmed pathological diagnoses. After applying these criteria, 110 C4A–C lesions were included in the study (Figure 1).

This study received approval from the Institutional Review Board of Kyungpook National University Chilgok Hospital (IRB No. 2024-10-022).

### 2.2. Data Collection

In this study, a comprehensive dataset was collected to evaluate both the clinical and imaging characteristics of patients undergoing breast ultrasonography. Clinical variables included patient age, the indication for ultrasonography (screening versus diagnostic), and presenting symptoms, facilitating stratification based on the initial clinical assessment. Additionally, imaging-related parameters were systematically documented, including the lesion type on ultrasound (mass versus non-mass) and breast density category, as both factors influence diagnostic performance and lesion detectability.

For the reference standard, the pathologic ground truth was classified into benign, atypical, or malignant categories based on biopsy or surgical pathology results. These three groups were first analyzed and compared separately. After this initial comparison, the atypia and malignant groups were combined into a “non-benign” category and evaluated against the benign group to further assess diagnostic discrimination.

AI-assisted image analysis was performed using CadAI-B^®^ for breast (CadAI-B v.2.1.2, BeamWorks Inc., Daegu, Republic of Korea), a deep learning-based CAD system designed for breast ultrasonography. For each lesion, CadAI-B^®^ automatically generated an AI-assigned BI-RADS category and provided a probability of malignancy (POM) as a continuous score ranging from 0 to 1. Subsequently, these AI-derived outputs were used to evaluate the diagnostic performance of the AI system. While the system is designed for real-time inference, representative static images were used for each case in this retrospective study to ensure a standardized evaluation consistent with the pathological ground truth.

The AI system uses deep convolutional neural networks (CNNs) trained in a weakly supervised manner [13,25]. In contrast to the traditional supervised learning requiring pixel-level annotations, this model learns to differentiate malignant, benign, and normal images by automatically prioritizing suspicious regions. It generates a relevance map, called CadAI-Map, which is a visualization of the spatial attention map of the CNN, providing pixel-level abnormality scores normalized from 0 to 1, alongside the POM and AI-assigned BI-RADS category. In this map, pixels with values approaching 1 are visualized in red, indicating a higher spatial correlation with malignant features (Figure 1). By processing every ultrasound frame in real time, the CadAI-Map assists clinicians in detecting suspicious regions and performing immediate risk assessment during the examination.

To provide clinically decision support, the POM of CadAI-B was mapped to the likelihood of malignancy in the ACR BI-RADS Atlas through a formal non-linear calibration process. This mapping function was optimized using an independent tuning dataset to align the model’s output distribution with the empirical distribution of histopathological malignancy and expert-assigned labels. Under this calibrated framework, a CadAI-Score of 0, reflecting the absence of any detected suspicious findings, was mapped to BI-RADS category 1. Cases where a lesion was identified but the CadAI-Score remained above 0 and below 3 were classified as category 2. Subsequently, scores ranging from 3 to 10 were designated as category 3. For suspicious findings, scores ranging from 10 to 50, 50 to 80, and 80 to 95 were mapped to categories 4A, 4B, and 4C, respectively, while scores of 95 or higher were classified as category 5.

To assess the performance of the AI system, its classifications were compared to the ground truth, defined as the final pathological diagnosis obtained from core needle biopsy or surgical excision. Diagnostic performance metrics—including sensitivity, specificity, positive predictive value, negative predictive value, and overall accuracy—were calculated based on whether the AI classified a lesion as suspicious for malignancy (BI-RADS ≥ C4) compared to the confirmed pathology. This approach facilitated a quantitative assessment of the ability of the AI system to differentiate benign, malignant, and atypical lesions in the study cohort.

In the study, both radiologist-interpreted BI-RADS categories and AI-generated BI-RADS assessments derived from an AI–based breast ultrasound analysis system were included. Collecting dual interpretations—expert human readings and AI-assisted evaluations—enabled direct comparison of diagnostic concordance, discordance patterns, and potential performance differences between radiologists and the AI system across diverse patient subgroups.

### 2.3. Breast Density

Breast density was evaluated using mammographic images analyzed with Lunit INSIGHT MMG (Lunit, Seoul, Republic of Korea), an AI-assisted software approved for mammography. All mammograms were obtained using dedicated 3D digital breast imaging equipment (Selenia^®^ Dimensions^®^; Hologic, Marlborough, MA, USA), while density classification followed the ACR BI-RADS breast composition categories (A–D). Lunit INSIGHT MMG automatically calculated the breast density category for each mammogram, and the results were reviewed and confirmed by board-certified breast specialists. Density was determined using mammograms acquired within 3 months of the ultrasound examination to ensure consistency between mammographic breast composition and the sonographic evaluation of each lesion.

Breast density on mammography was classified based on the fourth edition of the BI-RADS density categories [26]. The ACR BI-RADS breast composition categories were defined as follows: category B (scattered areas of fibroglandular density), category C (heterogeneously dense), and category D (extremely dense) [26]. Category A was not observed in this study; therefore, analyses were performed using categories B, C, and D.

### 2.4. Statistical Analysis

The primary outcomes of this study were sensitivity and specificity, calculated using the final pathological diagnosis (benign versus non-benign) as the reference standard. Based on these baseline indicators, additional diagnostic performance indices were derived, including positive predictive value (PPV), negative predictive value (NPV), and overall accuracy. As secondary outcomes, these performance measures were further stratified based on breast density categories to evaluate differences in diagnostic performance across density levels.

All collected variables were summarized as mean ± standard deviation (SD) for continuous variables and as counts with percentages for categorical variables. Sensitivity, specificity, PPV, and NPV were calculated using BI-RADS C4A as the diagnostic threshold, with lesions classified as positive or non-positive (benign versus atypical or malignant) for analytical purposes. Sensitivity, specificity, PPV, NPV, and accuracy were calculated using pathology as the reference standard. Exact 95% confidence intervals were estimated using the Clopper–Pearson binomial method to account for small subgroup sample sizes.

The unit of analysis was each lesion classified as BI-RADS C4. For patients with multiple lesions, only the lesion interpreted as C4 on imaging was selected for evaluation, and this lesion alone was analyzed using the AI-CAD system.

## 3. Results

The mean age of the 110 patients was 47.0 years (SD ±11.9). Among them, 75 patients (68.2%) underwent breast ultrasonography for screening purposes, while the remaining patients were evaluated for abnormal findings detected on other imaging modalities. Most patients (82.7%, n = 91) were asymptomatic at presentation; 15 patients (13.7%) presented with palpable breast nodules, and 3 patients (2.7%) presented with nipple discharge.

Among the 110 lesions, 90 (81.8%) were classified as C4A, 18 (16.4%) as C4B, and 2 (1.8%) as C4C. Overall, 78 lesions (70.9%) were benign, 7 (6.4%) were atypical, and 25 (22.7%) were malignant. For analysis, atypical and malignant lesions were grouped into a non-benign category and compared to the benign group. Within the benign category, fibrocystic change was the most common diagnosis, accounting for 22 lesions (20.0%), followed by benign phyllodes tumor in 3 lesions (2.7%). Among atypical lesions, atypical papilloma was the most common observed (4 cases, 3.6%). In the malignant group, invasive ductal carcinoma was the predominant subtype, identified in 16 cases (14.5%) (Table 1). Of the 78 benign lesions, 42 (53.8%) were classified as BI-RADS category 1–3, while 36 (46.2%) were classified as ≥C4 using AI ultrasound. Among the 32 non-benign lesions, only 6 (18.8%) were categorized as BI-RADS 1–3, representing benign lesions (Supplementary Table S1). Based on radiologist interpretation, 78 cases (70.9%) were classified as BI-RADS 4 and subsequently confirmed as benign on pathology, indicating that biopsies may have been unnecessary in these cases. In contrast, when AI-based BI-RADS assessment was considered, the number of benign cases classified as BI-RADS 4 or higher decreased to 36 cases (32.7%), suggesting a substantial reduction in potentially unnecessary biopsies (Supplementary Table S2).

Regarding ipsilateral breast density, 18 lesions (16.4%) were classified as ACR BI-RADS category B, 40 (36.4%) as category C, and 52 (47.2%) as category D. Across all density groups, non-benign lesions (atypical and malignant) demonstrated consistently higher POM than the benign lesions. The POM was highest in category B and relatively lower in categories C and D. Overall concordance with the histopathologic ground truth was 60.9%. Concordance increased progressively from density category B to C and D (B: 50.0%; C: 57.5%; D: 67.3%). Additionally, a dichotomized analysis comparing low-density (A–B) and high-density (C–D) breasts demonstrated higher concordance in the high-density group (A–B: 50.0% vs. C–D: 63.0%) (Table 2).

Across the 110 BI-RADS category 4 lesions included in this study, the distribution of disease categories varied based on breast density. In density B, benign lesions accounted for the majority (11/18, 61.1%), followed by malignant lesions (6/18, 33.3%) and atypical lesions (1/18, 5.6%). In category C, benign lesions accounted for 28 cases (70.0%) and malignant lesions for 9 cases (22.5%). A similar pattern was observed in category D, with benign lesions representing 39 cases (75.0%), while atypical and malignant lesions represented 3 cases (5.8%) and 10 cases (19.2%), respectively (Table 3).

**Figure 2:**
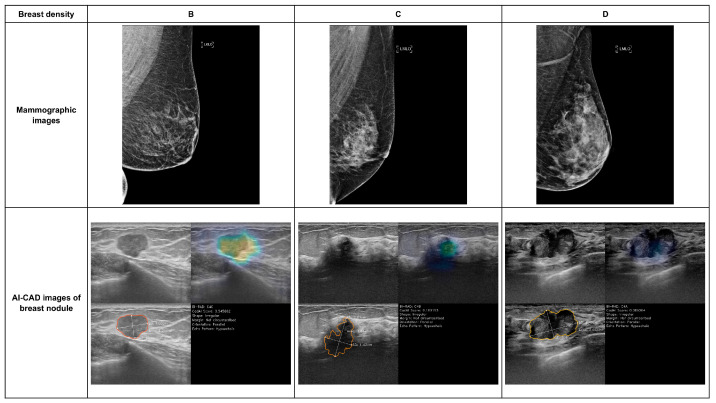
Mammographic images and AI-CAD images of breast nodule according to the breast density.

**Figure 3:**
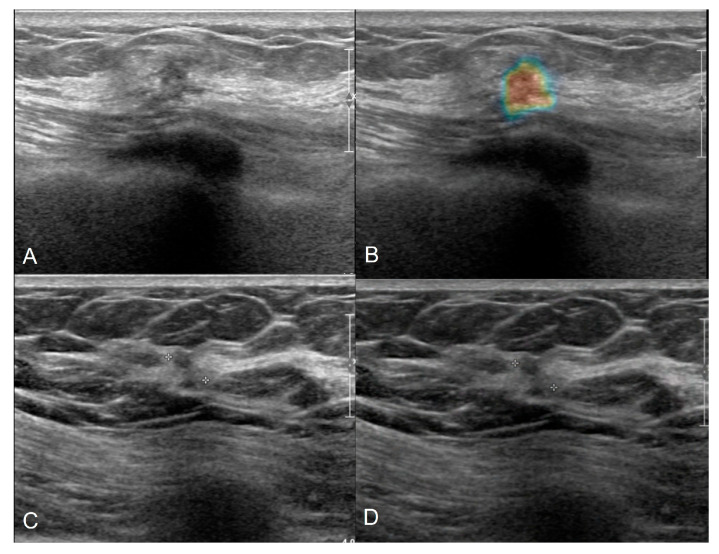
Ultrasound B-mode images show a lesion in a 65-year-old woman with mammographic density category C (**A**) with an AI true-positive assessment predicting a 62% probability of malignancy (C4C) (**B**), and a lesion in a 77-year-old woman with mammographic density category B (**C**) with a false-negative assessment predicting a probability of malignancy < 1% (C2) (**D**).

**Figure 4:**
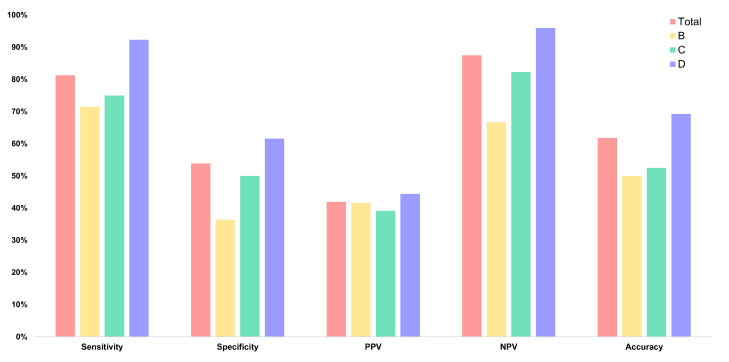
Comparison of diagnostic performances according to the breast density (B–D). PPV, positive predictive value; NPV, negative predictive value.

Overall diagnostic performance for the total cohort demonstrated a sensitivity of 81.3% and an NPV of 87.5%. In contrast, the overall specificity reached only 53.8%, and the PPV was 41.9%. The overall accuracy was 61.8%. The diagnostic performance of AI-assisted ultrasound generally increased with higher breast density (Figure 2 and Figure 3). In category B, the sensitivity, specificity, PPV, NPV, and accuracy were 71.4%, 36.4%, 41.7%, 66.7%, and 50.0%, respectively. In category C, sensitivity was 75.0%, specificity 50.0%, PPV 39.1%, NPV 82.4%, and accuracy 52.5%. In the density D group, all diagnostic performance metrics reached their highest values (Supplementary Figure S1). The sensitivity, specificity, PPV, and NPV were 92.3%, 61.5%, 44.4%, and 96.0%, respectively. The accuracy was 69.2%, representing the highest performance compared to the density B and C groups (Figure 4; Table 4).

## 4. Discussion

In this study, the diagnostic performance of AI-CAD ultrasound for BI-RADS category 4 breast lesions was evaluated across different breast density categories, indicating conditions closely aligned with real-world clinical application. A key finding of this study is the consistent variation in AI diagnostic performance across the breast density categories. As breast density increased, the sensitivity, specificity, PPV, NPV, and overall accuracy demonstrated a clear upward trend, with the highest values observed in the density D patient group. This finding suggests that AI-CAD ultrasound may delineate the lesion morphology and echogenicity more effectively in high-density breasts, potentially because lesion borders appear in a more uniform and well-defined pattern in higher-density tissue than in lower-density tissue.

In contrast, the density B group demonstrated lower specificity and PPV, indicating a greater tendency toward false-positive classifications. This reduced specificity may be attributed to the heterogeneous acoustic background created by intermixed fatty and fibroglandular tissue. In such transitional breast composition, normal anatomical structures—including fat lobules, stromal interfaces, and focal connective tissue—can generate hypoechoic or irregular sonographic appearances that mimic true lesions. Compared with predominantly fatty or uniformly dense breasts, density B exhibits greater textural variability, which may increase false-positive classifications for both human readers and AI-based models. These findings suggest that background tissue heterogeneity, rather than absolute breast density alone, plays a critical role in influencing specificity in ultrasound-based AI systems.

In addition, concordance between histopathologic diagnosis and AI-based BI-RADS classification increased with breast density. The density D group showed a higher concordance rate than the density B group, indicating that AI may identify lesion morphology more reliably in high-density breast tissue. This density-related improvement in concordance suggests that greater background uniformity in dense breasts may facilitate more consistent feature extraction and, consequently, enhanced diagnostic performance.

Although atypia is not classified as frank malignancy from a pathological perspective, atypical breast lesions are widely recognized as having significant malignant potential and are associated with an increased risk of subsequent carcinoma. In routine clinical practice, such lesions require histologic confirmation and often prompt surgical excision or close surveillance, similar to the management pathway for malignant lesions. Therefore, from a diagnostic and clinical decision-making standpoint, atypia represents a category that cannot be managed as benign. In the present study, we grouped atypia together with malignant lesions as a non-benign category to better reflect real-world clinical workflows focused on determining the need for biopsy and further intervention. This classification strategy allowed us to evaluate diagnostic performance in a manner that is clinically meaningful, emphasizing the distinction between lesions that require invasive confirmation or treatment and those that can be safely managed without biopsy.

Another significant consideration is the relevance of these findings to the clinical characteristics of Asian women. Approximately 50–70% of Asian women have dense breasts [17,18,19], with breast cancer developing approximately 10 years earlier than in Western women [20,21,22,23]. These demographic and epidemiologic factors support the use of ultrasound-based examinations as a key clinical tool. Furthermore, this study demonstrates that AI-based ultrasound achieves particularly high diagnostic performance in Asian patients with dense breasts. For example, an AI-based ultrasound was developed in a previous study to classify glandular tissue components in the breast, showing promising diagnostic results [27]. The findings suggest that further improvements could be achieved if AI-based models are specifically designed and calibrated to account for breast density.

Several AI-based breast ultrasound systems have been reported to assist radiologists by improving diagnostic consistency and reducing unnecessary biopsies. Most prior systems primarily focused on binary benign–malignant classification or overall diagnostic accuracy across mixed BI-RADS categories [3,14,23,27,28]. In contrast, the CadAI-B system evaluated in this study was specifically assessed in BI-RADS C4 lesions, the most clinically challenging category, and further analyzed according to breast density. This targeted approach allows CadAI-B to provide more clinically actionable support at the biopsy decision threshold, rather than general lesion classification. The observed density-dependent performance suggests that CadAI-B may offer particular advantages in dense breasts, where lesion interpretation is often more difficult in routine ultrasound practice. This study has some limitations. First, as a single-center, retrospective study, the sample size was relatively small, particularly for C4B and C4C lesions, potentially limiting detailed subtype analyses. Second, AI evaluation was performed using static images, which may differ from real-time AI analysis in clinical practice. Third, breast density was assessed using mammography rather than directly evaluating tissue shadow patterns inherent to ultrasound. Therefore, differences in the concept of breast density may exist between the two imaging modalities. Finally, only the performance of AI was evaluated based on a single interpretation against histopathologic results, while the potential synergistic effects of combined radiologist and AI interpretations in the actual diagnostic process were not assessed. Finally, breast density in this study was defined based on mammography, whereas the AI model was trained and evaluated using ultrasound images. Because mammographic density does not always directly correspond to background echogenicity on ultrasound, this modality mismatch may have indirectly influenced AI performance and should be considered when interpreting density-stratified results.

Nevertheless, this study evaluated AI-CAD ultrasound performance across breast density levels with a specific focus on BI-RADS 4 lesions, a category associated with substantial diagnostic uncertainty. The results indicate that AI-CAD may be clinically useful as a decision-support tool for radiologists, particularly in dense breasts common among Asian women, by helping to identify BI-RADS C4 cases that may be safely downgraded and managed without immediate biopsy. Future multicenter studies incorporating real-time clinical workflows are needed to validate these findings and establish density-aware strategies for AI-assisted ultrasound interpretation.

## 5. Conclusions

AI-CAD ultrasound demonstrated density-dependent performance in BI-RADS C4 lesions, with improved diagnostic metrics in denser breasts. The system may function as an adjunctive tool in clinical practice, particularly for Asian women who have a high prevalence of dense breasts, by supporting radiologists in refining biopsy decisions. Further prospective, multicenter studies are needed to validate these findings and establish density-aware clinical implementation strategies.

## Figures and Tables

**Table 1:** Clinical characteristics of patients had ACR BI-RADS category 4 breast lesions.

Variables		N = 110
Age (mean ± SD)		47.0 ± 11.9
Reason for examination (n, %)	Screening	75 (68.2)
	Diagnosis in other images	35 (31.8)
	Symptom	3 (2.7)
Symptom	None	91 (82.7)
	Breast nodule	15 (13.7)
	Nipple discharge	3 (2.7)
	Nipple eczema	1 (0.9)
ACR BI-RADS category	C4A	90 (81.8)
	C4B	18 (16.4)
	C4C	2 (1.8)
Ground truth	Benign	78 (70.9)
	Fibrocystic change	22 (20.0)
	Stromal fibrosis	14 (12.7)
	Papillary lesion	10 (9.1)
	Sclerosing adenosis	10 (9.1)
	Fibroadenoma	7 (6.4)
	Benign phyllodes tumor	3 (2.7)
	Complex sclerosing lesion	2 (1.82)
	Others	10 (9.1)
	Atypia	7 (6.4)
	Atypical papilloma	4 (3.6)
	Atypical ductal hyperplasia	2 (1.8)
	Atypical lobular hyperplasia	1 (0.9)
	Malignancy	25 (22.7)
	Invasive ductal carcinoma	16 (14.5)
	Ductal carcinoma in situ	6 (5.5)
	Invasive lobular carcinoma	2 (1.8)
	Invasive carcinoma, unknown type	1 (0.9)

**Table 2:** Comparison of disease classification and concordance of AI results with pathology according to breast density.

Breast Density	N (%)	Benign		Non-Benign		Concordance of AI Results with Pathology (B vs. C vs. D)	Concordance of AI Results with Pathology (A–B vs. C–D)
		N (%)	POM *	N (%)	POM		
B	18 (16.4)	11 (61.1)	0.2849	7 (38.9)	0.5034	50.0%	50.0%
C	40 (36.4)	28 (70.0)	0.1485	12 (30.0)	0.3485	57.5%	63.0%
D	52 (47.2)	39 (75.0)	0.1836	13 (25.0)	0.4104	67.3%	
Total	110 (100.0)	78 (10.9)	0.1853	32 (29.1)	0.4075	60.9%	60.9%

* Probability of malignancy.

**Table 3:** Disease category of AI-based BI-RADS according to breast density.

Breast Density		B			C			D			Total
BI-RADS		C1/2	C3	≥C4	C1/2	C3	≥C4	C1/2	C3	≥C4	
Disease category (n, %)	Benign	2 (100.0)	2 (50.0)	7 (58.3)	10 (90.9)	4 (66.7)	14 (60.9)	12 (92.3)	12 (100.0)	15 (55.6)	78 (70.9)
	Atypia	0 (0.0)	1 (25.0)	0 (0.0)	0 (0.0)	1 (16.7)	2 (8.7)	1 (7.7)	0 (0.0)	2 (7.4)	7 (6.4)
	Malignancy	0 (0.0)	1 (25.0)	5 (41.7)	1 (9.1)	1 (16.7)	7 (30.4)	0 (0.0)	0 (0.0)	10 (37.0)	25 (22.7)
	Total	2	4	12	11	6	23	13	12	27	110

**Table 4:** Comparison of diagnostic performances with BI-RADS category by AI results according to breast density.

Breast Density	Sensitivity (%) (95%-CI)	Specificity (%) (95%-CI)	Positive Predictive Value (%) (95%-CI)	Negative Predictive Value (%) (95%-CI)	Accuracy (%) (95%-CI)
B	71.4 (29.0–96.3)	36.4 (10.9–69.2)	41.7 (15.2–72.3)	66.7 (22.3–95.7)	50.0 (26.0–74.0)
C	75.0 (42.8–94.5)	50.0 (30.6–69.4)	39.1 (19.7–61.5)	82.4 (56.6–96.2)	57.5 (40.9–73.0)
D	92.3 (64.0–99.8)	61.5 (44.6–76.6)	44.4 (25.5–64.7)	96.0 (79.6–99.9)	69.2 (54.9–81.3)
Total	81.3 (63.6–92.8)	53.8 (42.0–65.3)	41.9 (29.5–55.2)	87.5 (74.8–95.3)	61.8 (52.1–70.9)

## Data Availability

The datasets generated and/or analyzed during the current study are not publicly available. However, they are available from the corresponding author upon reasonable request.

## Supplementary Materials

The supporting information can be downloaded at: https://www.mdpi.com/article/10.3390/cancers18030536/s1.
