# Supplementary material for: Effect of a Real-Time Artificial Intelligence-Assisted Ultrasound System on BI-RADS C4 Breast Lesions Based on Breast Density
Source: Cancers (Basel). 2026 Feb 6;18(3):536. doi: 10.3390/cancers18030536 (PMC12897095; doi:10.3390/cancers18030536)

Supplementary Table S1. Probability of malignancy and BI-RADS category by AI ultrasound based on disease classification.

Disease classification		N (%)	POM* (mean)	ACR BI-RADS by AI ultrasound (n, %)					
				Benign		Non-benign			
				C1/2	C3	C4A	C4B	C4C	C5
Benign	Benign	78 (70.9)	0.1852	24 (92.3)	18 (81.8)	9 (69.2)	13 (59.1)	14 (53.8)	0
Non-benign	Atypia	7 (6.4)	0.2607	1 (3.8)	2 (9.1)	1 (7.7)	1 (4.5)	2 (7.7)	0
	Malignancy	25 (22.7)	0.4486	1 (3.8)	2 (9.1)	3 (23.1)	8 (36.4)	10 (38.5)	1 (100.0)
Total		110	0.2499	26	22	13	22	26	1

*Probability of malignancy

Supplementary Table S2. Disease categorization based on radiologist-assigned ACR BI-RADS assessment.

ACR BI-RADS	Benign	Atypia	Malignancy	Total
C4A	73 (81.1%)	6 (6.7%)	11 (12.2%)	90 (81.8%)
C4B	5 (27.8%)	1 (5.6%)	12 (66.7%)	18 (16.4%)
C4C	0 (0.0%)	0 (0.0%)	2 (100.0%)	2 (1.8%)
Total	78 (70.9%)	7 (6.4%)	25 (22.7%)	110 (100.0%)

Supplementary Figure S1.

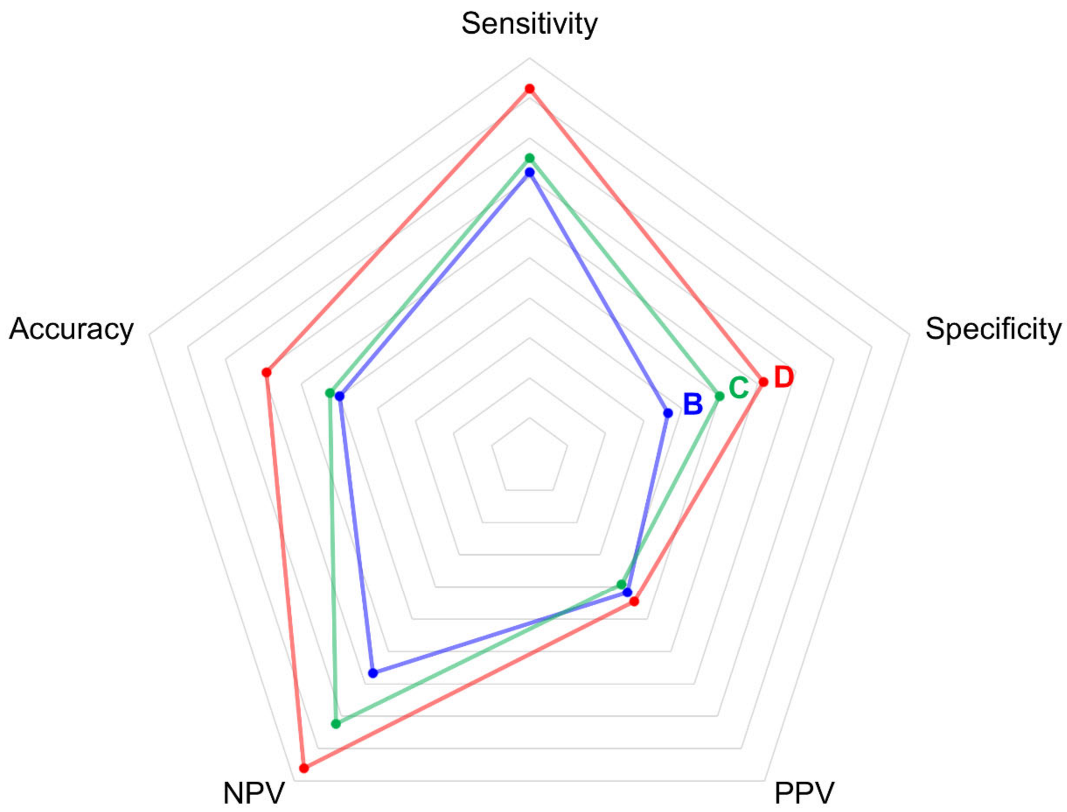

Supplement: Supplementary file 1 [file cancers-18-00536-s001.zip › cancers-4114462-supplementary.pdf]
